# Corticotropin-Releasing Factor and Toll-Like Receptor Gene Expression Is Associated with Low-Grade Inflammation in Irritable Bowel Syndrome Patients with Depression

**DOI:** 10.1155/2016/7394924

**Published:** 2016-07-12

**Authors:** Song Jizhong, Wang Qiaomin, Wang Chao, Li Yanqing

**Affiliations:** ^1^Department of Gastroenterology, Qilu Hospital, Shandong University, Jinan 250012, China; ^2^Department of Gastroenterology, Anhui Provincial Hospital, Hefei 230001, China

## Abstract

The mechanism of low-grade inflammation in irritable bowel syndrome (IBS) is unclear; our research concentrates on the involvement of the corticotropin-releasing factor (CRF) and Toll-like receptor (TLR) gene expression in the process of low-grade inflammation in IBS patients with depression. This study suggests more IBS patients are presenting with the states of depression and anxiety. IBS patients with depression have shown a lower grade inflammatory response and an imbalance of the inflammatory response. CRF1, CRF2, TLR2, and TLR4 in IBS patients with depression are significantly higher than those without depression and controls. Thus, activation of the CRF-TLR associated pathways produces an inflammatory reaction, which can concurrently affect the digestive tract and the CNS and induce the corresponding digestive and psychiatric symptoms.

## 1. Background

Irritable bowel syndrome (IBS) is a functional gastrointestinal disease characterized by abdominal pain or discomfort and is associated with altered bowel habits and (or) changes in stool characteristics. Stress is one of the main causes of the disease, and so, childhood maltreatment, divorce and long-term work pressure, may induce or aggravate the symptoms of IBS [1]. Recent studies show that stress can lead to abnormal gastrointestinal motility and visceral hypersensitivity through the modulation of the Hypothalamic-Pituitary-Adrenocortical (HPA) axis: that is, the abnormal expression of corticotropin-releasing factor (CRF) may be one of the main reasons [2]. The increase of CRF expression can lead to mental symptoms, such as anxiety and depression [3]. Increasing evidences indicate that some IBS patients often expressed mental disorders such as anxiety or depression [4]. Thus, the above findings show that CRF may contribute to IBS symptoms as well as to psychiatric symptoms, indicating that there may be a common pathophysiological process underlying IBS and psychiatric disorders.

The inflammation processes, such as respiratory and intestinal infections, can downregulate mood, induce abdominal distension, or lead to a decrease of appetite. However, how peripheral inflammation acts on the central nervous and digestive system is still unclear. Neal et al. [5] found that it is not so rare that intestinal infection induces IBS. A study focusing on postinfectious IBS found that inflammation exists in intestinal mucosa [6]. Furthermore, another study found that non-postinfectious IBS also had low-grade inflammatory response, such as the increase of IL-1, IL-6, and TNF [7]. A previous study found that intestinal mucosal barrier was damaged and the intestinal permeability was increased, thereby leading to immune activation in IBS patients under stress [8]. Dowlati et al. [9] also revealed that there was immune activation and inflammatory response in patients with depression, but the mechanism of the induction of this inflammatory reaction was not jet clarified. A study found that IBS and psychiatric symptoms might overlap and that psychiatric disorders may make IBS symptoms difficult to control [10]. Thus, whether IBS and psychiatric symptoms in these patients have the inflammation as the common denominator is worth investigating.

In this study, we assessed the expressions of inflammatory cytokines, CRF, and Toll-like receptors (TLR) using RT-PCR to elucidate the possible association between intestinal inflammation and depression in IBS patients.

## 2. Methods

### 2.1. Patients

IBS patients and healthy controls were invited to join this study consecutively at the outpatient digestive department in Anhui Provincial Hospital, from October 2014 to December 2015. One hundred two age- and sex-matched healthy control subjects were selected from subjects who underwent physical examination and questionnaire survey and had negative results. All subjects were asked to complete the Hamilton Depression Scale (HAMD) with 24 items and the Hamilton Anxiety Scale (HAMA) with 14 items questionnaires. IBS was diagnosed according to the Rome III criteria [11]. All patients underwent physical examination, detailed history collection, and gastroscopy and colonoscopy to rule out organic diseases.

### 2.2. HAMD and HAMA Questionnaire

IBS patients with HAMD score ≥7 were defined as having IBS with depression (DP-IBS). IBS patients with HAMD score <7 were defined as having IBS without depression (NDP-IBS). In accordance with the matching principle of the balance, we again randomly selected 60 cases from the 102 healthy controls using a random number table. Peripheral blood samples were taken from all IBS patients and 60 selected cases, and mRNA expressions of IL-6, IL-10, CRF1, CRF2, and TLR4, TLR2 in the peripheral blood samples were detected by RT-PCR.

This study was approved by the Anhui Provincial Hospital Ethics Committees (2014-54). All participants gave their written informed consents.

### 2.3. RNA Extraction and Reverse Transcription

Red cell lysis (BL503A, Biosharp, China) liquid was precooled and then added to the samples at a volume ratio of 1 : 5. After 10 minutes of centrifugation at 4°C and 3000 rpm, the upper layer was discarded. Total RNA was extracted using the standard Trizol (10296-010, Invitrogen, USA) method. The quantitative and qualitative analyses of the RNA were performed using an ultraviolet spectrophotometer. The total RNA was then reversely transcribed into cDNA using reverse Transcriptase kit (Invitrogen 1685474, USA) by BIOMETRA Tprofessional.

### 2.4. Quantitative Real-Time Polymerase Chain Reaction (qRT-PCR)

The amplified DNA was analyzed by the comparative Ct method using *β*-actin as a reference gene. The primer and probe sets for IL-6 (F: ACCC CCAT TAAA TATA GGAC TGGA; R: AGTT CATA GCTG GGCT CCTG) (Sangon Biotech China), IL-10 (F: CGAG ATGC CTTC AGCA GAGT; R: CGCC TTGA TGTC TGGG TCTT) (Sangon Biotech China), CRF1 (F: GTGC CCCA TTTC AGGT TCG; R: GAAG TAGT TGTA GGCG GCTG T), CRF2 (F: CAAC CTCT CAGG TCCC TACT CC; R: GATC TTTG AGGC CCAC GTCC) (Sangon Biotech China), TLR2 (F: TCCT GCTA AGAG ACTC CTCT GT; R: AACA AGTT TTGG GGAG TGCC) (Sangon Biotech China), and TLR4 (F: GCTC GGTC AGAC GGTG ATAG; R: TGTG TGGT TTAG GGCC AAGT) (Sangon Biotech China). The qRT-PCR was performed under the following amplification conditions: total volume of 20 *μ*L, initial incubation at 50°C/2 min followed by denaturation at 95°C/10 min, and then 45 cycles at 95°C/15 sec and at 60°C/1 min. Analysis of relative gene expression data was done by using the 2^−ΔΔCt^ Method [12].

### 2.5. Statistical Analysis

SPSS 17 statistical software was used for statistical analysis. The measurement data of normal distribution are expressed as the mean ± SD, independent Student's *t*-test was used in the two-group comparison, and the variance analysis was used to compare the three groups. The count data were expressed by percentage and the chi square test. The *P* value < 0.05 was taken as statistically significant.

## 3. Results

(1) A total of 102 IBS patients and 102 healthy controls entered the present study. In patients with IBS, 43.13% (*n* = 44) were diagnosed with diarrhea-predominant (IBS-D) IBS, 29.41% (*n* = 30) were constipation-predominant (IBS-C) IBS, 15.69% (*n* = 16) were IBS-M (mixed diarrhea and constipation), and 11.76% (*n* = 12) were IBS-A (alternating stool pattern). The HAMD score of IBS group was 19.19 ± 16.998, which was higher than that in healthy control group (3.20 ± 2.903) (*t* = 8.966, *P* < 0.01). The HAMA score of IBS group was 17.250 ± 14.296, which was also higher than that in healthy control group (5.86 ± 3.162) (*t* = 7.837) (Table 1).

(2) A total of 58 cases (56.9%) got a HAMD score ≥7 (DP-IBS), and 44 cases (43.1%) got a HAMD score <7 (NDP-IBS). IBS patients living in urban setting, married, or with a higher education were more likely to express depressive symptoms (Table 2).

(3) HAMD scores of 102 IBS patients were positively correlated with IL-6 mRNA expression (*r* = 0.455, *P* < 0.01) and negatively with IL-10 mRNA expression (*r* = 0.028, *P* = 0.667).

(4) Comparison of IL-6 mRNA and IL-10 mRNA expression was made between DP-IBS, NDP-IBS, and the control group.

**Figure 1:**
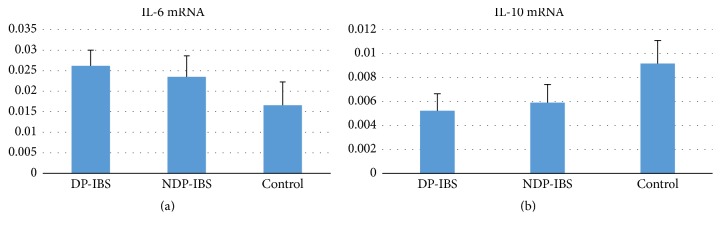
(a) IL-6 mRNA expression between DP-IBS, NDP-IBS, and the control group (0.026 ± 0.004, 0.023 ± 0.005, and 0.017 ± 0.006, resp., *F* = 53.077, *P* < 0.01). IL-6 mRNA levels in DP-IBS and NDP-IBS patients were significantly higher than those in the control group. IL-6 mRNA levels in DP-IBS patients were significantly higher than that in NDP-IBS patients (*P* < 0.05). (b) IL-10 mRNA expressions in DP-IBS and NDP-IBS patients were significantly lower than those in the control group (0.0092 ± 0.0019, 0.0059 ± 0.0015, and 0.0052 ± 0.0014, resp., *F* = 58.249, and *P* < 0.05). IL-10 mRNA level in DP-IBS is lower than NDP-IBS, but there was no statistical significance (*P* > 0.05).

IL-6 mRNA expressions in DP-IBS and NDP-IBS patients were significantly higher than those in the control group (0.026 ± 0.004, 0.023 ± 0.005, and 0.017 ± 0.006, resp., *F* = 53.077, *P* < 0.01), and DP-IBS patients had a higher IL-6 mRNA level than that of NDP-IBS patients, *P* < 0.05. (Figure 1(a)). IL-10 mRNA expressions in DP-IBS and NDP-IBS patients were significantly lower than those in the control group (0.0092 ± 0.0019, 0.0059 ± 0.0015, and 0.0052 ± 0.0014, resp., *F* = 58.249, *P* < 0.05). IL-10 mRNA level in DP-IBS is lower than NDP-IBS, but without statistical significance (*P* > 0.05) (Figure 1(b)).

(5) Comparison of expressions of CRF1 mRNA and CRF2 mRNA was made between DP-IBS, NDP-IBS, and the control group.

**Figure 2:**
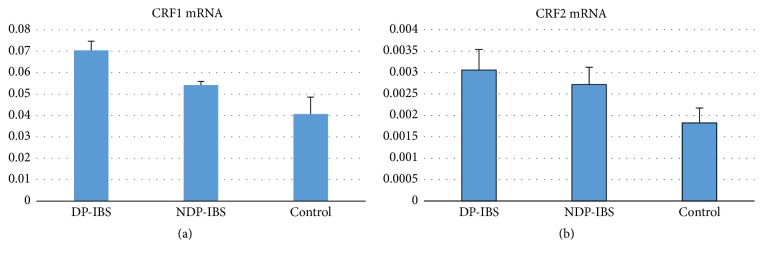
(a) CRF1 mRNA in DP-IBS, NDP-IBS, and control group (0.070 ± 0.004, 0.054 ± 0.002, and 0.041 ± 0.008, resp., *F* = 12.047, *P* < 0.01). CRF1 mRNA expression levels in DP-IBS and NDP-IBS patients were significantly higher than those in the control group and in DP-IBS patients were significantly higher than in NDP-IBS patients (*P* < 0.05). (b) CRF2 mRNA expressions in DP-IBS, NDP-IBS, and control group (0.0031 ± 0.0005, 0.0027 ± 0.0004, and 0.0018 ± 0.004, resp., *F* = 83.726, *P* < 0.01). CRF2 mRNA levels in DP-IBS and NDP-IBS patients were significantly higher than those in the control group (*P* < 0.05).

CRF1 mRNA and CRF2 mRNA levels in DP-IBS and NDP-IBS patients were significantly higher than those in the control group (CRF1: 0.070 ± 0.004, 0.054 ± 0.002, and 0.041 ± 0.008, resp., *F* = 12.047, *P* < 0.01; CRF2: 0.0031 ± 0.0005, 0.0027 ± 0.0004, and 0.0018 ± 0.004, resp., *F* = 83.726, *P* < 0.01). CRF1 mRNA and CRF2 mRNA levels in DP-IBS patients were significantly higher than those of NDP-IBS patients, *P* < 0.05 (Figures 2(a) and 2(b)).

(6) Comparison of TLR2 mRNA and TLR4 mRNA expression levels between DP-IBS, NDP-IBS, and the control group.

**Figure 3:**
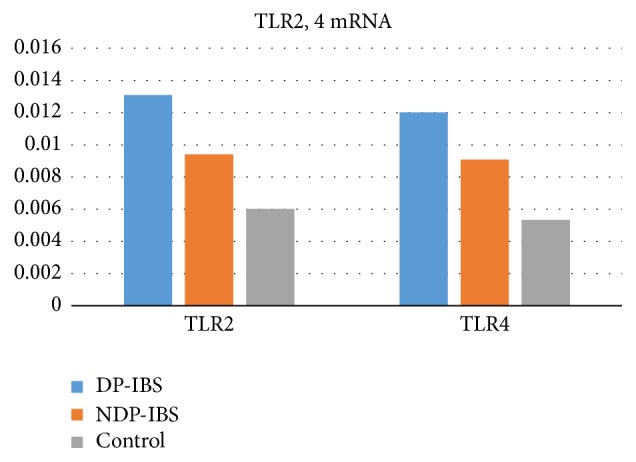
TLR2 and TLR4 mRNA expressions in DP-IBS, NDP-IBS, and the control group (0.013 ± 0.002, 0.009 ± 0.003, and 0.006 ± 0.002, resp., *F* = 5.018, *P* = 0.008; 0.012 ± 0.002, 0.009 ± 0.001, and 0.005 ± 0.001, resp., *F* = 12.015, *P* < 0.01). The TLR2 mRNA levels in DP-IBS and NDP-IBS patients were significantly higher than those in the control group, and those in DP-IBS patients were significantly higher than those in NDP-IBS patients (*P* < 0.05). TLR4 mRNA levels in DP-IBS and NDP-IBS patients were significantly higher than those in the control group, and those in DP-IBS patients were significantly higher than those of NDP-IBS patients (*P* < 0.05).

TLR2 mRNA and TLR4 mRNA levels in DP-IBS and NDP-IBS patients were significantly higher than those in the control group (0.013 ± 0.002, 0.009 ± 0.003, and 0.006 ± 0.002, resp., *F* = 5.018, *P* = 0.008; 0.012 ± 0.002, 0.009 ± 0.001, and 0.005 ± 0.001, resp., *F* = 12.015, *P* = 0.000), and those in DP-IBS patients were significantly higher than those in NDP-IBS patients (*P* < 0.05) (Figure 3).

## 4. Discussion

IBS is characterized by abdominal pain and abnormal bowel movement pattern. It affects approximately 10–20% of the general population. The pathogenesis remains unclear, but accepted mechanisms involve interaction between triggering environmental factors, abnormal gastrointestinal motility, and disturbed visceral sensory perception. Increasing evidence supports coexistence of IBS with anxiety, depression, and other psychiatric symptoms [4], but it is difficult to determine the cause and effect relationship. However, most scholars believe these exist as comorbidity [10]. We found that the HAMD and HAMA scores of the IBS group were higher than those of the healthy control group. The result shows that more than half of IBS patients expressed anxiety and depression symptoms. The data is roughly consistent with those provided by the American Gastroenterological Association Clinical Practice Committee [13]. Using HAMD score of 7 as a cut-off value, 102 cases of IBS patients were divided into DP-IBS (58 cases, 56.9%) and NDP-IBS (44 cases, 43.1%), with depressive symptoms as an additional parameter to further determine differences between DP-IBS, NDP-IBS, and the healthy control group. In general, when comparing these three groups, only urban life, married status, and higher education in the IBS patients increased the risk for depressive symptoms. This suggests that the increasing demands of everyday life, such as a fast-paced work lifestyle, as well as the accompanying chronic mental strain may increase the incidence of depression in IBS patients. Despite the impact of age and gender on the onset of IBS itself [14], it does not increase the risk of IBS associated with depressive symptoms. Thus, chronic stress is a common cause of IBS and depression symptoms.

Neal et al. [5] were the first to propose that patients with intestinal infection are prone to develop IBS with a low-grade inflammatory response. There is growing evidence to support immune activation and increase of IL-1, IL-6, and TNF-*α* levels in IBS patients [7, 15]. Depression is also harmful to human health and the emotional underpinnings of major diseases, where the incidence increases significantly, often coexisting with multiple somatic diseases [16]. Previous studies have focused more on the nervous system, but more recent studies have shown that depression is associated with low-grade inflammatory reaction. Thus, some scholars believe that depression is an inflammatory disease [9, 17, 18]. In a clinical study, it was reported that there was a significant increase in sera TNF-*α* and IL-1*β* levels and IL-1*β*/IL-10 ratio in 42 depressive patients [19]. Thomson et al. [20] used LPS to induce TLR to produce an inflammatory reaction in mice, which resulted in a significant increase in interferon in the central nervous system (CNS), which suggests that chronic inflammatory diseases cause a CNS inflammatory response, thereby resulting in depression and anxiety symptoms. Anti-TNF-*α* treatment for Crohn's disease can significantly improve the patient's symptoms of depression, which is further evidence of chronic inflammation leading to psychiatric symptoms [21]. There is increasing literature to support the concept that IBS and depression are both present with low-grade inflammation. The present study shows that IL-6 mRNA expression was positively correlated with HAMD scores, that IL-6 mRNA in DP-IBS was higher than that in controls, and that IL-10 mRNA level was significantly lower. This suggests that there were a low-grade inflammatory response and an imbalance of the inflammatory response in DP-IBS patients. Overall, low-grade inflammatory reactions are common in IBS and depression symptoms.

CRF is made up of a 41-amino-acid neuropeptide acting through two receptors. CRF1 and CRF2 are key hormones of the HPA axis under stress. CRF and the CRFR are widely distributed in the CNS and peripheral tissues and play an important regulatory role in physiological homeostasis and stress responses. In general, the CRF1 receptor is the principal receptor mediating the stress response, whereas the CRF2 receptor modulates the effects of CRF1 signal transduction [22]. Binder et al. [23] had suggested that CRF is involved in the stress response through multiple pathways. Interaction between the CRF1 receptor and an adverse environment can increase the risk of stress related mental disorders. A study using conditional CRF1 receptor knockout mice showed that anxiety- and depression-related symptoms are specific to the activation of the CRF1 receptor in the limbic forebrain regions [24]. Notably, persistent elevation of CSF CRF concentration in symptomatically improved depressed patients is associated with early relapse of depression [25]. Posserud et al. [26] also observed that CRF levels were significantly increased during stress in IBS patients. A study using Wistar-Kyoto rats showed a significant increase in CRF1 and alteration in the affective component of visceral pain modulation [27]. A single-center, randomized, double-blind, three-period crossover study showed that a CRF-R1 antagonist could relieve abdominal pain, fear, and impaired learning in 11 women with IBS [28]. Above all, it showed that CRF plays an important role in inducing symptoms of IBS and depression under stress.

CRF also acts locally as a proinflammatory mediator. Yuan et al. [29] provided anatomical support for a role of CRF-1 signaling in modulating the immune-inflammatory process in a UC study involving the colonic mucosa of ulcerative colitis patients. Astressin, a nonselective CRF receptor antagonist, can abolish the effect of LPS-stimulated IL-1 and IL-6 release and downregulate threshold of visceral sensation in rats [30]. Urocortins are members of the CRF family of neuropeptides that were found to modulate immunosignaling via CRF receptor in a rat model of colitis [31, 32]. It was also reported that CRF is an important proinflammatory mediator in IBS and depression symptoms [27]. Under psychological stress, CRF triggers increase in intestinal paracellular permeability via mast cell-dependent release of TNF-*α* and proteases [33]. Evidence indicates that stress through the brain-gut axis may cause intestinal barrier dysfunction, mainly via the systemic and peripheral release of corticotropin-releasing factor [34]. Therefore, the impaired intestinal mucosal barrier via CRF may be a prerequisite for low-grade inflammation.

TLRs are critical pattern recognition molecules of the innate immune system. In general, TLR signaling in health contributes to homoeostatic functions, such as when microbes break through the physical barrier of the body, such as skin or mucous membrane. TLRs can identify the foreign agent and activate the body to produce an immune cell response. More studies have shown that increased TLRs activate an inflammatory process in the gut, such as in inflammatory bowel disease and intestinal infection [35]. Recently, studies have begun to focus on the relationship between TLRs and IBS. Belmonte et al. [36] reported that the IBS-mixed subgroup had significant upregulation of TLR2 and TLR4 in the colonic mucosa and increased expression of the mucosal proinflammatory cytokines IL-8 and IL-1*β*. McKernan et al. [37] also observed significant increases in the TLR3, TLR4, and TLR5 mRNA levels in both the distal and proximal colonic mucosa of MS rats compared with controls, which suggests that innate immune receptor expression may be changed in the gastrointestinal tract of animals with stress-induced IBS-like symptoms. In addition, accumulating evidence suggests that TLRs are involved in the pathophysiology of major depressive disorder. Hung et al. [38] examined TLR1~9 mRNA expression level in peripheral blood and its relationship with the psychopathology of major depressive disorder. Multiple linear regression analysis revealed that TLR4 is an independent risk factor relating to severity of major depression. Fifty-six patients were treated with antidepressant which relieved their depressive symptoms and decreased their levels of TLR1, TLR2, TLR4, and TLR6 mRNAs [39]. Kéri et al. [40] found that TLR4 mRNA signaling is upregulated in first-episode major depression, and increased IL-6 and C-reactive protein result in a moderate inflammatory reaction. The present study shows that TLR2 and TLR4 mRNAs in DP-IBS patients are higher than in the control group, which indicates that TLR participates in the inflammation reaction in IBS and depression. Further study is needed to determine if CRF is directly adjusting TLR level. Tsatsanis et al. [41] suggest that CRF peptides may mediate the upregulation of TLR4 via the CRF-2 receptor and produce LPS-induced proinflammatory cytokines from macrophages. Wang et al. [42] also found that CRH augments LPS-induced cytokine secretion in human trophoblast cells via upregulation of TLR4. A review by Subbannayya et al. [43] indicates that CRH activates TLR4 via the protein kinase A pathway, which produces proinflammatory cytokines.

## 5. Conclusions

IBS patients during stress release CRF which leads to the damage of the intestinal mucosa barrier. TLR2 and TLR4 activation produces an inflammatory reaction, which can concurrently affect the digestive tract and the CNS, and induces the corresponding digestive and psychiatric symptoms.

## Figures and Tables

**Table 1:** Comparison of HAMD and HAMA scores in 102 patients with IBS and control group.

		IBS patients	Controls	*χ* ^2^ value	*P* value
Years	<20	27	19	0.1796	0.180
	20~50	56	58	0.0800	0.778
	>50	19	25	1.043	0.307
Gender	Male	63	57	0.729	0.393
	Female	39	45	0.729	0.393

				*t* value	

HAMD		19.19 ± 16.998	3.20 ± 2.903	8.966	*P* < 0.01
HAMA		17.250 ± 14.296	5.86 ± 3.162	7.837	*P* < 0.01

**Table 2:** Comparison between the baseline characteristics of DP-IBS and NDP-IBS patients.

		DP- IBS	NDP-IBS	*χ* ^2^ value	*P* value
Years	<20	20	17	0.187	0.666
	20~50	23	18	0.016	0.0898
	>50	15	9	0.407	0.524
Gender	Male	21	23	2.633	0.105
	Female	37	21		
Domicile	Urban	39	27	5.904	<0.05
	Rural	19	17		
Marital status	Unmarried	21	10	2.149	0.143
	Married	31	32	30938	<0.05
	Widow	6	2	1.164	0.281
Socioeconomic status	Low	7	14	0.000	0.883
	Middle	28	23	0.160	0.689
	High	23	7	6.795	<0.01
